# Uncovering high rates of unsafe injection equipment reuse in rural Cameroon: validation of a survey instrument that probes for specific misconceptions

**DOI:** 10.1186/1477-7517-8-4

**Published:** 2011-02-07

**Authors:** Mbah P Okwen, Bedes Y Ngem, Fozao A Alomba, Mireille V Capo, Savanna R Reid, Ebong C Ewang

**Affiliations:** 1 Health Sector, Netherlands Development Organization (SNV), No 10 Cowstreet, Bamenda,PO Box 5069, Bamenda,NWR, Cameroon; 2 Department of Statistics, Bali District Health Services, No 1 Lamsi Street, BaliPO Box 42, BaliNWR, Cameroon; 3 Bali District Health Services, No 1 Lamsi Street, BaliPO Box 42, BaliNWR, Cameroon; 4 Water, Sanitation and Hygiene Sector, Netherlands Development Organization (SNV) No 10 Cowstreet, Bamenda,PO Box 5069, Bamenda,NWR, Cameroon; 5 School of Community Health Sciences, University of Nevada at Las Vegas, 431 Sunburst Dr., Henderson, NV 89002, USA; 6 District Hospital Bali, No 1 Lamsi Street, BaliPO Box 42, BaliNWR, Cameroon

## Abstract

**Background:**

Unsafe reuse of injection equipment in hospitals is an on-going threat to patient safety in many parts of Africa. The extent of this problem is difficult to measure. Standard WHO injection safety assessment protocols used in the 2003 national injection safety assessment in Cameroon are problematic because health workers often behave differently under the observation of visitors. The main objective of this study is to assess the extent of unsafe injection equipment reuse and potential for blood-borne virus transmission in Cameroon. This can be done by probing for misconceptions about injection safety that explain reuse without sterilization. These misconceptions concern useless precautions against cross-contamination, i.e. "indirect reuse" of injection equipment. To investigate whether a shortage of supply explains unsafe reuse, we compared our survey data against records of purchases.

**Methods:**

All health workers at public hospitals in two health districts in the Northwest Province of Cameroon were interviewed about their own injection practices. Injection equipment supply purchase records documented for January to December 2009 were compared with self-reported rates of syringe reuse. The number of HIV, HBV and HCV infections that result from unsafe medical injections in these health districts is estimated from the frequency of unsafe reuse, the number of injections performed, the probability that reused injection equipment had just been used on an infected patient, the size of the susceptible population, and the transmission efficiency of each virus in an injection.

**Results:**

Injection equipment reuse occurs commonly in the Northwest Province of Cameroon, practiced by 44% of health workers at public hospitals. Self-reported rates of syringe reuse only partly explained by records on injection equipment supplied to these hospitals, showing a shortage of syringes where syringes are reused. Injection safety interventions could prevent an estimated 14-336 HIV infections, 248-661 HBV infections and 7-114 HCV infections each year in these health districts.

**Conclusions:**

Injection safety assessments that probe for indirect reuse may be more effective than observational assessments. The autodisable syringe may be an appropriate solution to injection safety problems in some hospitals in Cameroon. Advocacy for injection safety interventions should be a public health priority.

## Introduction

The most common invasive health care procedures in Cameroon are medical injections, which have the potential to transmit blood-borne infections such as HIV, HBV and HCV when injection equipment is unsafely reused[1,2]. Multiple use of single use devices is common practice due to cost constraints in developing countries[3]. In Cameroon the cost of medical care is borne by a patient population living in rural poverty-one third are below the international poverty line of $1.25 per day[4]. Single use devices such as disposable syringes are not designed to withstand heat sterilization for safe reuse. From a public health perspective, unsafe reuse is not cost-saving[5]. The costs of nosocomial infections resulting from unsafe reuse are borne by the patients, who may face stigmatizing illness without knowing how they became infected.

Shortage of supply is not the only explanation for unsafe reuse. Many practices that endanger patient safety are related to health workers' misconceptions about infection control. Probing for these misconceptions may shed light on the prevalence of risky behaviors. Since 1992, more than 600 iatrogenic HBV and HCV outbreaks have been traced to reuse of injection equipment in countries with a low prevalence of these viruses[6]. Important misconceptions identified behind these outbreaks include the beliefs that (1) it is safe to reuse a syringe after changing the needle, (2) it is safe to reuse a needle or syringe on the same patient, re-entering a multi-dose vial or saline bag with a used needle or syringe, and (3) it is safe to reuse a needle or syringe when accessing an IV port separated from the patient by intervening lengths of IV tubing or the presence of heparin locks or valves. In some instances providers change the needle to reuse the syringe on the same patient only, but the multidose vial may nevertheless become contaminated under these circumstances. These specific practices are sometimes referred to as "indirect reuse," as opposed to overt reuse of needles and syringes without any of these useless precautions.

An experimental assessment of two of these precautions-changing the needle to reuse the syringe, and reusing only to access an injection port separated from the patient by a length of IV tubing protected by heparin locks or valves-found neither precaution prevented blood contamination of the syringe[7]. Only if IV extension tubing is used will the third injection site (furthest from the patient) remain uncontaminated. The extension tubing itself cannot be used on multiple patients safely, and one instance of such reuse has already led to a major patient notification and outbreak investigation in the U.S. (Broward General Medical Center, Fort Lauderdale, Florida). The expense of providing each patient with extension tubing renders the precaution potentially more expensive than using a new needle and syringe for every injection. All forms of indirect reuse have been linked to HBV or HCV transmission in outbreak investigations[8]. Reuse of the syringe after changing the needle has been linked to rapid HIV transmission in an outbreak investigated in Russia in 1989[9]. An outbreak of HIV traced to syringe reuse to flush an IV line at a dialysis clinic in Egypt in 1993 led to 64 infections in patients, or 32% of all HIV infections in Egypt at that time[10].

Problems with health governance in public hospitals in Cameroon also contribute to unsafe practices such as reuse without sterilization. For example, a healthcare worker may illegally collect money from a patient to buy syringes or surgical materials and then economize on the number of syringes or materials bought, to maximize personal gain. Health workers earn $400 per month after taxes, $55 less than the cost of supporting an average family of five. In addition, most hospital sharps waste disposal systems are substandard (authors' personal observation). The presence of loose sharps waste contributes to reuse of equipment for the same patient or use of leftovers from another patient. The community does not participate in deciding how or where medical waste is disposed, and dumping of sharps waste in open spaces creates additional risk.

The connection between problems with health governance and problems with infection control is not unique to Cameroon. The U.S. Centers for Disease Control (CDC) has supported investigations that identified unsafe injections among the important transmission routes in three large iatrogenic HIV outbreaks in Romania,[11-15] Kazakhstan,[16] and Kyrgyzstan[17]. In 2010 the CDC investigated the exceptionally high prevalence of HIV in the rural town of Jalal Pur in Pakistan, an anomaly discovered at a mobile HIV screening in 2009. This investigation determined that over many years unsterile medical injections acted as a bridge between the concentrated HIV epidemic in high risk groups and the general population[18]. All of these outbreaks were traced to corrupt practices by health workers who either reused equipment without precautions or practiced extortion by charging patients for supplies that were not new.

The World Health Organization recommends an observational assessment protocol (Tool C) for evaluating medical injection safety in developing countries[19]. An injection safety assessment team observed 92 injections at 77 health care facilities in Cameroon in 2003 using Tool C. These observers found that 100% of injections were given with a needle and syringe taken from a new, sealed package[20]. Indirect reuse was hardly observed, as 98% of reconstitutions were performed with a needle and syringe taken from a new, sealed package. These findings are a seeming underestimate of actual reuse rates. Observational patient safety performance assessments are problematic because the presence of a visiting observer influences performance and adherence to the standard precautions. This research methods problem is usually referred to as the Hawthorne effect.

The authors have commonly observed unsafe reuse in the Northwest Province of Cameroon. Patients who make an informal payment for a new needle and syringe may receive an injection with a used syringe. The Cameroon Ministry of Public Health launched a campaign against hospital corruption in 2007, 'Hopital Sans Corruption.' This campaign attempted to educate patients not to pay for any medical services directly to health care workers or without the issuance of hospital receipts. This campaign was not welcomed by healthcare workers and it soon died down as they argued that these illegal practices had no impact on the patients' well being (authors' personal observation). An initial study revealed that the primary consequence of poor governance at district hospitals was poor quality of service. Interest in health governance reform is ongoing. The district health service of Bali requested the assistance of the Netherlands Development Organization in 2010 to evaluate the concrete effects of poor governance in terms of unsafe injection practices.

The purpose of this survey of infection control practices in maternity wards and outpatient wards in all public hospitals in two rural health districts was to assess the risk to patients from injection equipment reuse. These health districts are located in the Northwest Province of Cameroon, a region noted within the country for higher standards of patient care. Survey methods that probe for misconceptions about injection safety were compared with records indicating how many syringes and needles are supplied to these hospitals. We also assessed sharps waste disposal at these hospitals. The rates of blood-borne virus transmission through unsafe medical injections in these hospitals were estimated using a model. Data on injection frequency were taken from the national Demographic and Health Survey conducted in 2004[21].

## Methods

A total of 69 (of 98) health workers at fifteen hospitals were interviewed on their own infection control practices. In assessing injection safety, we investigated four types of reuse: (1) reuse of the syringe and needle, (2) reuse of the needle after changing the syringe, (3) reuse of the syringe after changing the needle, and (4) reuse of a needle or syringe to flush a patient's catheter. These questions (see Additional file 1) were selected to capture the most common types of reuse identified in blood-borne virus outbreaks[8]. Data collectors were drawn from among health staff working at the hospitals investigated who were highly motivated to improve injection safety. They participated in a six hour workshop in sensitive interviewing techniques. The numbers of needles and syringes supplied to these hospitals were collected to investigate whether supplies were inadequate.

The number of HIV, HBV and HCV infections that result from unsafe medical injections can be estimated from the number of injections performed (n), the probability of unsafe reuse (p_r_), the probability that reused injection equipment had just been used on an infected patient (p_v_), the size of the susceptible population (p_s_), and the transmission probability of each virus in an unsafe medical injection (p_t_)[22].

(1) $$\text{Incidence}=\text{n}\times {\text{p}}_{\text{r}}\times {\text{p}}_{\text{v}}\times {\text{p}}_{\text{s}}\times {\text{p}}_{\text{t}}$$

The average adult in Cameroon receives 2.4 medical injections each year according to the 2004 Demographic and Health Survey (DHS)[21]. The publically available DHS data set shows that 11.3% of these injections were given to HIV positive patients. The prevalence of HBV among blood donors in Cameroon ranges from 6-16% and the prevalence of HCV in blood donors ranges from 0.8-3.9%[23,24]. No other recent estimates of HBV prevalence are available, but these figures may underestimate population prevalence. Estimates of HCV prevalence in Cameroon range up to 13.8%, with lower prevalence in blood donors and young women (1.8-1.9%) due to a marked age cohort effect[25-28].

The WHO estimates the probability of transmission in an unsafe medical injection is 1.2% for HIV, 6% for HBV (but 30% if the source patient is a carrier of the hepatitis B e antigen), and 1.8% for HCV[22]. An estimated 15% of HBV positive adults in Cameroon are carriers of the hepatitis B e antigen[29]. The WHO estimate of the probability of HIV transmission per unsafe medical injection is the midpoint of a range of estimates (0.3-2.3%) developed from studies of accidental needle injuries in health workers[30]. Alternatively, a nosocomial HIV outbreak infecting more than 1,000 children in Romania suggested transmission rates of 3-7%[30]. Recently it has been argued that rinsing or wiping injection equipment eliminates this transmission risk in medical settings[31]. However, a needlestick accident involves only the insertion of a needle. The plunger is not depressed in an accidental stick and the contents of the syringe are not injected. A calculation of the difference in administered inoculum volume between insertion of a needle and injection of the contents of the syringe shows that this difference offsets the reduction in inoculum volume achieved by rinsing a needle and syringe between uses[32].

## Results

In total 44% of health workers reported practicing some form of unsafe injection equipment reuse. The most common practice is reuse of the syringe after changing the needle (36%). Several health workers practiced more than one type of unsafe reuse. Only 2% of health workers reported they would reuse a needle and syringe on another patient, but 39% would reuse either the needle or the syringe. In total 13% would reuse injection equipment to flush a patient's catheter (Table 1).

**Table 1:** Self-reported injection equipment reuse in Northwest Province of Cameroon, 2010.

Type of reuse	Providers reporting reuse	
None	39	(56%)

Syringe and needle	1	(2%)

Syringe only	17	(25%)

Needle only	2	(3%)

Only to flush a catheter	2	(3%)

Syringe or needle	1	(2%)

Syringe/to flush a catheter	7	(10%)

Total	69	

Health workers' self-reported behavior agreed with the records of injection supplies ordered at some hospitals. This suggests that many of those who reported reusing injection equipment did so routinely. Either the overall syringe reuse rate in the first health district was lower than the percentage of health workers who reported sometimes reusing syringes, or many health workers reused syringes when there was no shortage of supply. Not all hospitals where reuse is practiced had purchasing policies that created a shortage of syringes. (Table 2)

**Table 2:** Supplies of syringes and needles in one health district, January to December 2009.

Hospital	Syringes and needles	Needles	Butterfly needles and canullars^1^	Discrepancy between needles and syringes^2^	Self-reported syringe reuse
Urban public hospital	2852	700	300	24%	33%

Private hospital	22160	0	405	0%	0%

Rural public hospital 1	0	100	0	-	0%

Rural public hospital 2	100	0	50	0%	50%

Rural public hospital 3	611	0	310	0%	25%

Rural public hospital 4	927	200	250	21%	25%

District Hospital	3300	2200	2517	67%	66%

**Total**	**29950**	**3200**	**3832**	**11%**	**36%**

1. Used to secure IV lines.

2. (Total needles consumed, excluding butterfly needles and canullars-total syringes consumed) ÷ Total syringes consumed = Total syringes reused, assuming all needles consumed were used.

Supply records suggest at least 11% of injections given in these hospitals were performed with reused syringes in 2009. Under this assumption, unsafe injection practices may have led to 14-336 HIV infections, 248-661 HBV infections and 7-114 HCV infections between January and December 2009. Actual numbers may be higher or lower depending on the injection equipment reuse practices of other health care providers in these districts. These estimates are conservative, missing at least two types of reuse practiced at these hospitals. The number of unsafe injections administered through intravenous lines or with reused needles could not be estimated from the available data.

Only two of the fourteen hospitals used a standard incinerator for medical sharps waste. All other hospitals practiced open dumping and irregular burning. Most burned medical waste more than once a month.

## Discussion

In Cameroon, the quest to attain the millennium development goals has been greatly hindered, indirectly, by poor governance issues at public facilities and intractable problems with infection control[33]. This survey revealed that injection equipment reuse is exceedingly common in Cameroon. Little effort is made to sterilize syringes between uses on multiple patients. Purchasing practices have resulted in a shortage of syringes at some hospitals. This is an unacceptable approach to dealing with budget shortfalls. Sharps waste management is also substandard. Self-reported injection equipment reuse may be more reliable than observational injection safety assessments, provided the survey instrument probes for specific types of injection equipment reuse that health workers may mistakenly believe to be safe. If data collectors are not recruited from among the respondents' colleagues they cannot reasonably expect to obtain honest answers in a face-to-face interview. Under these circumstances an anonymous self-administered paper and pencil questionnaire completed in a one-on-one interview is recommended (see Additional file 1). This approach has succeeded both in injection safety research and in research investigating informal payments for health services[34,35].

Many health staff participating in the survey expressed a desire to change these unsafe practices. Even those implicated in corrupt practices were unhappy with the situation. One respondent in the present survey noted that he feels nauseous when he 'eats' (spends) the money he collects in illicit payments for unsafe injections. This remark is consistent with previous research showing that informal payments for health services are not good for morale[36]. Nevertheless, informal payments continue to be seen as critically important compensation for health staff in low wage countries where salary payments may be irregular[37]. Measures to eliminate unsafe injection equipment reuse must account for the staying power of these practices. Transparency International ranks the health sector 9^th ^among the 20 sectors most affected by corruption in Cameroon in 2006[38]. Research into informal payment systems has shown that improving wages alone is not an adequate prevention-unless penalties are in force, corrupt practices will continue[39]. Some African countries now restrict the importation of syringes that do not have reuse prevention features that engage automatically (see Additional file 2). Additional file 3 presents a detailed description of corruption problems in hospitals in Cameroon and proposed measures to improve health governance.

The practice of reusing injection equipment contributes to millions of serious infections worldwide each year. The WHO estimates that 5% of HIV infections, 32% of hepatitis B infections and 40% of hepatitis C infections result from unsafe medical injections in the developing world[22]. The high prevalence of hepatitis C virus in older age groups in Cameroon dates to mass injection campaigns carried out in the colonial era with unsafe injection practices[40]. In the effort to eradicate sleeping sickness, leprosy, and syphilis with intravenous injections, hepatitis C transmission was so intense that in the most affected age cohorts prevalence exceeded 50%[41]. Recent research into the viability of HIV recovered from syringe washes in Cameroon suggests that historic unsafe reuse of syringes used for phlebotomy and IV injections and lack of testing of blood for transfusion may have fueled the massive expansion of HIV subtype CRF_02 in this region[42]. Ongoing research is exploring the hypothesis that these practices also contributed to SIV human infection 50 to 60 years ago.

The role of unsafe medical injections in Africa's HIV epidemic has been debated since the beginning of HIV epidemiology[43]. When only data on prevalent HIV infection were available, it made sense to assume that injections were associated with HIV infection because those with advanced HIV and AIDS needed curative injections. Most reports on the association often observed between receiving medical injections and recent HIV infection note that this data is also difficult to interpret. Fourteen prospective studies conducted in Africa looked at curative and birth control injections as causes of recent HIV infection[44-57]. The median fraction of HIV transmission attributed to medical injections in these studies is 18%. These associations are often discounted because little was done to control for overlapping sexual exposures or the need for medical injections to treat early HIV disease (seroconversion illness).

More recently, Brewer, Roberts and Potterat have shown that antenatal tetanus injections and phlebotomy injections are associated with prevalent HIV infection in ten countries sub-Saharan Africa, including Cameroon[58]. This study is well controlled for demographic and sexual confounders. This analysis excluded women who had previously tested for HIV and who may have been referred to antenatal care for screening to enroll in services for the prevention of mother-to-child HIV transmission. The combined adjusted odds ratio for exposure to these two punctures is 1.29 (95% CI 1.08-1.54). As 80% of women who had been pregnant within the past 5 years in Cameroon had been exposed, this risk factor explains 19% of HIV infections in women with young children in Cameroon. A possible confounder in this association between HIV and tetanus and phlebotomy injections is the receipt of other unsafe injections during attendance at the antenatal clinic. Some women may have stopped by for antenatal services because they were attending the clinic for other curative services.

Like research into HIV origins, controversies in contemporary blood-borne HIV epidemiology in Africa are tied up in a narrative of blame[59]. The practice of unsafe reuse raises ethical concerns for the public health community, and the language of injustice has been invoked to advocate for reform[60]. Health workers are bound by the Principles of Non-Maleficence and Beneficence to take every possible measure to protect vulnerable patients from healthcare-associated infections[61]. We are certain that the realm of the possible now includes the prevention of HIV transmission from patient to patient in countries with generalized AIDS epidemics. In 2004, 15 predominately Western authorities in HIV epidemiology wrote to *Lancet *that the effect of the elimination of unsafe injections would be inconsequential to the AIDS epidemic in Africa[31]. Five years later, 27 predominately African scientists and public health officials signed a joint statement of the research agenda concerning unsafe health care in Africa listing several flaws in this argument[62]. They called for higher quality research into blood-borne HIV and an end to tolerance for unsafe health care practices.

Resource constraints continue to hinder the fight against HIV in Africa, and the moral distress this causes health workers is a strain on the profession[63]. An ethos of triage has been invoked in the field of HIV research in the interests of shutting down debate over blood-borne HIV[31]. A timely interest in pursuing a zero tolerance policy for nosocomial HIV in high prevalence countries is nevertheless possible. Observational injection safety assessments across Africa have shown widespread endorsement of single use policies[64]. Underlying problems with indirect reuse make health workers uncomfortable and can certainly be changed. Related problems with health governance are already targets for reform[65].

Despite single use guidelines in the World Health Organization's best practices for safe injections, injection equipment reuse is common practice where resources for injection safety training and infection control supervision are lacking. Indirect reuse, such as reusing a syringe after changing the needle, has been specifically reported in Burkina Faso, South Africa and Swaziland[66-68]. The same practices are still reported by a few injection providers in high income developed countries, including the U.S.[69,70] We hypothesize that other forms of indirect reuse can also be observed around the world. The misconceptions that lead to reuse without sterilization seem to arise from the structure and appearance of injection equipment and not from local traditions. We outline methods for assessing the relative importance of various specific misconceptions in Additional file 4.

In 2000, syringe reuse rates in Burkina Faso were comparable to contemporary reuse rates in the United States. This achievement stands in the face of widespread pessimism about the possibility of eliminating unsafe reuse in least developed countries. Burkina Faso has since adopted a national injection safety policy that restricts the importation of syringes that are not reuse prevention feature syringes that engage automatically, like the auto-disable syringe.

In 2001, the World Health Organization recommended the use of auto-disable syringes for all immunization injections in the Expanded Programme on Immunizations. For the first several years of this guideline, this recommendation did not cover reconstitution syringes. A more recent recommendation for autodisable syringe use issued by UNICEF applies to reconstitution syringes as well. The reconstitution syringe can contaminate a multi-dose vial if reused on the same patient when drawing more vaccine, and if the contaminated multidose vial is used again multiple subsequent patients can be infected. Viable HIV has been recovered from an experimentally contaminated medication vial, showing that an HIV outbreak may also occur if injection equipment is reused on the same patient when accessing a multi-dose vial or saline bag[71]. Multiple viral hepatitis outbreaks in the U.S. have been traced to this practice[8].

Importantly, the majority of medical injections given in Africa are not immunization injections. In the Democratic Republic of Congo, Nigeria, Tanzania and Uganda, reuse prevention feature syringes are now required for all medical injections. Like Burkina Faso, Tanzania now restricts the importation of syringes that do not have reuse prevention features that engage automatically.

Another WHO target for improving injection safety in developing countries is eliminating unnecessary medical injections. The overuse of injectable medicines in developing countries is often perceived as demand-driven, due to cultural beliefs that injections are more powerful than other forms of medication. But recent research has shown that injection providers overrate demand for injections and are mistaken to assume that all their patients wish to receive an injection. Overuse of injections is also institutionalized in national essential drug lists. There are 32 injectable drugs (excluding five anesthetic agents) on Cameroon's essential drug list (out of 149 medications). Of these injectable drugs, all but seven are available in oral formulations. Countries facing serious problems with injection equipment reuse could replace injectable drugs with their oral formulations on the national essential drug list to reduce risk. The transition to oral first-line treatment for malaria throughout sub-Saharan Africa has made important inroads in this direction.

Under the President's Emergency Plan for AIDS Relief the U.S. CDC will support and USAID will fund any iatrogenic HIV outbreak investigation in Africa if invited to support such an investigation by the Ministry of Health. However, at present resources for identifying outbreaks are limited in Africa. Surveillance for HIV infections in children with HIV negative mothers exists only in South Africa[72]. CDC officials have indicated an interest in launching an investigation at the point when unsafe injection practices are identified rather than limiting investigations to recognized clusters of epidemiologically linked infections[8]. Detection of outbreaks is difficult even where blood-borne virus surveillance exists. Routine case-investigations have shown 50% of persons interviewed do not report behavioral risk factors for acute hepatitis B and C in the U.S.[73] This finding suggests widespread undetected nosocomial transmission in a country with blood-borne virus surveillance and low levels of reuse. Problems in high prevalence countries may be quite serious without being obvious in the absence of surveillance.

Unfortunately programmatic funding for injection safety interventions under PEPFAR ended in 2010. Millions of dollars have been spent developing and supporting national injection safety policies in ten African countries under PEPFAR's Making Medical Injections Safer (MMIS) program. However, these interventions only indirectly addressed unsafe reuse, by promoting the standard that a new needle and syringe be removed from a new, sealed package for every injection for the patient to see. This standard was developed to empower patients. In many countries this training was supported with public education campaigns creating demand for safe injections. However, this standard does not involve the patient as an active observer if injections are given to inpatients through an IV line. Countries that achieved nearly 100% compliance with this standard using Tool C also showed less compliance during reconstitution. This discrepancy may arise from the persistent misconception that it is safe to reuse on the same patient. Although MMIS trained thousands of health workers in injection safety, they did not explicitly address the misconceptions that lead to indirect reuse.

The efficacy of MMIS interventions carried out so far has only been assessed using WHO Tool C[64]. Our findings suggest Tool C is an inconclusive measure of blood-borne virus transmission risk. Continued attention to injection safety in MMIS countries and programmatic interventions in other African countries are still needed. Advocacy to PEPFAR and other donor programs and research funding bodies should place due emphasis on stopping the reuse of syringes, inappropriate use of multidose vials, and reuse to access IVs. Injection safety assessments in developing countries that fail to observe reuse during formal visits should not engender a false sense of security about the safety of injection practices. Unsafe reuse is a sensitive behavior that has not yet been eradicated in the United States[70]. Injection equipment reuse is a possible threat to public health that warrants further investigation in most countries with a high prevalence of blood-borne viruses.

We do not join other authors in calling for a reallocation of research and prevention funds presently needed in the fight against sexual HIV transmission. We consider the positive evidence of a heterosexual HIV epidemic in Africa robust and uncontroversial. Much has been made of the null results of randomized control trials studying STD treatment as an HIV prevention measure[74,75]. Nevertheless, exposure to other STDs is reliably predictive of exposure to HIV in observational studies[76]. Randomized treatment trials have no bearing on this research finding. As resources for HIV prevention diminish, injection safety interventions need to be integrated into other health systems strengthening and health workforce development programs. They cannot be expected to compete for priority with sexual HIV prevention activities.

## Conclusions

The present survey, unlike a previous observational assessment of injection safety in Cameroon using WHO Tool C, detected high rates of injection equipment reuse without sterilization. Our approach to probing for unsafe reuse, a sensitive behavior that may be concealed from visiting observers, may be more effective than the WHO standard. Where data collectors cannot be enrolled from among local health staff and prepared in an intensive workshop to perform sensitive interviews, alternative strategies are needed. Anonymous written questionnaires probing for the common misconceptions that explain reuse without sterilization have succeeded in other settings in developing and high income developed countries.

The autodisable syringe may be an appropriate solution to injection safety problems in some hospitals in Cameroon. Corruption contributes to unsafe reuse in this health care system and may frustrate interventions that depend on voluntary behavior change. However, injection safety trainings may also have benefit, as providers readily admitted to unsafe practices. The Safe Injections Coalition based in the U.S. has developed multimedia injection safety training materials that specifically address the misconceptions that lead to reuse without sterilization, the "One and Only Campaign" video, signage and brochures http://www.oneandonlycampaign.org/. Patients should be taught to expect to see a new needle and syringe removed from a new, sealed package for every injection. Educational interventions in health systems where serious problems with corruption are directly linked to unsafe practices should be supported with institutional reforms to improve health governance. Implementation of these strategies will take time. A more immediate, administrative strategy to reduce risk is to replace injectable drugs with their oral formulations on the national essential drug list.

## Competing interests

The authors declare that they have no competing interests.

## Authors' contributions

MPO served as principal investigator for this study; he facilitated the development of questionnaires, choosing of study participants and training of data collectors. He also contributed to data collection, analyses and development of manuscript. BYN contributed to developing questionnaires and data collection. FAA contributed to developing questionnaires. MVC

supervised and proof read questionnaires before implementation; she also contributed to manuscript finalization. Also approved sponsor of the study, SNV. SRR contributed to literature review, developing questionnaires, statistical analysis, and development of manuscript. ECE

contributed to developing questionnaires, facilitating data collection and mobilizing staff to participate in study. All authors read and approved the final manuscript.

## Authors' information

MPO: This is a medical doctor in Cameroon; he has been practicing medicine and research in resource limited setting and research. He works as advisor and country focal point for health at the Netherlands Development Organisation (SNV). Related publications include: (1) Unsafe injections: A joint statement of the Research Agenda, International Journal of STD & AIDS; (2) Detection of a new sub genotype of HBV in Cameroon but not in neighbouring Nigeria, Clin Microb Infection March 2010. BYN: This is a mid wife nurse practicing nurse at Bali DHS; he has 6 years of experience in maternity and public health care in resource limited setting. His daily work includes taking deliveries, collecting DHS data and supervising staff in the health areas. FAA: He is the district medical officer at Bali Health District. MVC: She is the Portfolio coordinator at SNV North West region. She is a Water, Sanitation and Hygiene (WaSH) expert of Beninoise nationality currently doing advisory practice in Cameroon. SRR: She is a MPH student at the University of Nevada at Las Vegas. ECE: This is a medical doctor and chief medical officer at the district hospital in Bali. He has been in medical practice for about 10 years in resource limited settings in most parts he has been in position of medical director of district hospitals.

## Supplementary Material

Additional file 1 **A Patient Safety Assessment protocol that lays out both the procedures piloted in Cameroon and an alternative formulation for use as an anonymous questionnaire to be self-administered in a one-on-one interview**. These questions performed well in the field and are recommended to rapidly establish whether a risk of blood borne virus transmission exists at a given health care facility. Click here for file

Additional file 2 **A policy document from the Tanzania Food and Drugs Authority**. It describes the new injection safety policy in Tanzania, restricting the importation of syringes that do not have reuse prevention features that engage automatically. Click here for file

Additional file 3 **Hospital corruption in Cameroon**. A copy of a newsletter that details the findings and recommendations of recent investigations into hospital corruption in Cameroon. Click here for file

Additional file 4 **Questions for All Injection Providers**. An expanded injection safety assessment questionnaire informed by infection control compliance research that explores the reasons for unsafe injection equipment reuse. Click here for file
