# Supplementary material for: Uncovering high rates of unsafe injection equipment reuse in rural Cameroon: validation of a survey instrument that probes for specific misconceptions
Source: Harm Reduct J. 2011 Feb 7;8:4. doi: 10.1186/1477-7517-8-4 (PMC3041680; doi:10.1186/1477-7517-8-4)
Supplement: Additional file 1 — A Patient Safety Assessment protocol that lays out both the procedures piloted in Cameroon and an alternative formulation for use as an anonymous questionnaire to be self-administered in a one-on-one interview. These questions performed well in the field and are recommended to rapidly establish whether a risk of blood borne virus transmission exists at a given health care facility. [file 1477-7517-8-4-S1.DOC]

Background

Patient safety research is turning a corner both high income countries and African countries as the potential role for unsterile health care procedures to contribute to HIV transmission is more widely recognized.[[1]](#footnote-2) The existence of widespread misconceptions about injection safety that lead some providers to reuse without sterilization is an important discovery that public health officials are now moving to redress.[[2]](#footnote-3) Improved models of healthcare HIV transmission risks suggesting an increased role for unsterile injections in the epidemic have put patient safety on the AIDS research agenda in Africa.[[3]](#footnote-4),[[4]](#footnote-5)

Large nosocomial HIV outbreaks have been investigated in several low-prevalence countries over 1989-2009 (Russia, Romania, Libya, Kazakhstan, Kyrgyzstan, and Uzbekistan).[[5]](#footnote-6) Many of these outbreaks involved reuse of injection equipment without sterilization.^5^ In the rural town of Jalal Pur in Pakistan, unsterile medical injections have served as a bridge between the concentrated HIV epidemic in high risk groups and the general population over a period of many years, leading to exceptionally high HIV prevalence in the community, discovered in 2009.[[6]](#footnote-7) This year, the U.S. Centers for Disease Control (CDC) has launched a domestic injection safety campaign in response to multiple large hepatitis outbreaks, for the prevention of a widely feared HIV outbreak.[[7]](#footnote-8) The CDC has also pledged to support any African country in an outbreak investigation and patient safety intervention should the Ministry of Health approach the CDC for such support, and funding for these activities is provided under the President’s Emergency Plan for AIDS Relief (PEPFAR).[[8]](#footnote-9)

The role of unsafe medical injections in Africa’s HIV epidemic has been debated because the association often observed between receiving medical injections and recent HIV infection is difficult to interpret. Fourteen prospective studies conducted in Africa looked at curative and birth control injections as causes of recent HIV infection.[[9]](#footnote-10),[[10]](#footnote-11),[[11]](#footnote-12),[[12]](#footnote-13),[[13]](#footnote-14),[[14]](#footnote-15),[[15]](#footnote-16),[[16]](#footnote-17),[[17]](#footnote-18),[[18]](#footnote-19),[[19]](#footnote-20),[[20]](#footnote-21),[[21]](#footnote-22),[[22]](#footnote-23),[[23]](#footnote-24) The median fraction of HIV transmission attributed to medical injections in these studies is 18%. These latter studies may have been confounded by sexual exposures or the need for medical injections to treat early HIV disease (seroconversion illness), however.

In a more recent meta-analysis of ten Demographic and Health Surveys in African countries (Cameroon, Ethiopia, Ghana, Guinea, Kenya, Lesotho, Malawi, Rwanda, Senegal and Zimbabwe), Brewer, Roberts and Potterat have shown that tetanus injections and blood draw injections are significantly associated with prevalent HIV infection.[[24]](#footnote-25) Women who had previously tested for HIV were excluded from the analysis to avoid counting women referred for antenatal services (including tetanus and blood draw injections) because of a pre-existing HIV infection. The association explains 15-21% of HIV infections in women who have given birth within the last five years in these surveys.

The auto-disable syringe is recommended for all immunizations (including tetanus injections) by the World Health Organization – but this policy has not yet been adopted for reconstitution syringes used in immunizations, or for curative and blood draw injections. Immunizations in particular are often packaged in multidose vials, and if a reconstitution syringe re-enters a multidose vial after use on a patient, the vial and all medication in it must be discarded for the safety of other patients. In one outbreak, contamination of a single multidose vial during reconstitution led to HIV infections in four subsequent patients.[[25]](#footnote-26)

These patient safety assessment tools are intended to discover risks for HIV, HBV and HCV transmission in common health care procedures. Reuse without sterilization is often practiced in hospitals with working sterilization equipment and operational sterilization routines, because these practices arise from misconceptions about whether or not the equipment has really been contaminated by the previous procedure. Only rinsing reusable equipment is sometimes practiced even when autoclaves are available.

Although these practices are often a reflection of corruption, many health care providers will readily admit to them because they mistakenly believe the practices to be safe and acceptable. Interviews with injection providers are believed to be more informative than observations of injection practices, as most health workers will reach for a new needle and syringe while under formal observation. If possible, it is worthwhile to review records of supplies ordered by the hospital, to establish whether there is an imbalance between the number of needles and syringes used. This will indicate whether changing the needle to reuse the syringe is a rare practice or a routine.

This assessment instrument was developed in partnership between the University of Nevada at Las Vegas and the Netherlands Development Organization (SNV) office in Bamenda, Cameroon. These questionnaires have been piloted at public hospitals in Bali and Batibo Health Districts, North West Province, Cameroon. The current version was developed for the Network of African People Living with HIV for Southern African Region.

Questions for all injection providers:

1. In the past six months, have you changed the needle to reuse a syringe? ( Y / N )
2. In the past six months, have you changed the syringe to reuse a needle? ( Y / N )
3. In the past six months, have you reused a needle or syringe to access an IV line?

( Y / N )

1. In the past six months, have you reused a needle or syringe on the same patient, to get more medication from a multidose vial? ( Y / N )
2. In the past six months, have you reused a finger stick device? ( Y / N )
3. In the past six months, have you reused a pair of gloves while giving injections? ( Y / N )
4. If you reuse injection equipment, how do you prepare it for reuse?

________________________________________________________________________________________________________________________________________________________________________________________________________________________________________________________________________________________________

1. Have you been trained in injection safety? ( Y / N ) If yes, when? ___________________

Questionnaire for Injection Providers

(1) Would you reuse a syringe after changing the needle?

1. I often do this.
2. I sometimes do this.
3. I rarely do this.
4. I can think of some circumstances under which I would do this.
5. I would never do this.

(2) Would you reuse a needle or a syringe when accessing an IV port?

1. I often do this.
2. I sometimes do this.
3. I rarely do this.
4. I can think of some circumstances under which I would do this.
5. I would never do this.

(3) Would you reuse a syringe on the same patient?

1. I often do this.
2. I sometimes do this.
3. I rarely do this.
4. I can think of some circumstances under which I would do this.
5. I would never do this.

(4) Would you reuse a needle on the same patient?

1. I often do this.
2. I sometimes do this.
3. I rarely do this.
4. I can think of some circumstances under which I would do this.
5. I would never do this.

(5) Would you reuse a needle or syringe after rinsing or soaking it in warm water?

1. I often do this.
2. I sometimes do this.
3. I rarely do this.
4. I can think of some circumstances under which I would do this.
5. I would never do this.

(6) Would you reuse a needle or syringe after heat sterilization?

1. I often do this.
2. I sometimes do this.
3. I rarely do this.
4. I can think of some circumstances under which I would do this.
5. I would never do this.

(7) Would you reuse a fingerstick device?

1. Usually I reuse fingerstick devices after wiping or rinsing off visible blood.
2. I rarely reuse fingerstick devices after wiping or rinsing off visible blood.
3. I never reuse fingerstick devices without cleaning them with a disinfectant effective against HIV, HBV and HCV or a 1:10 bleach solution.
4. I never reuse fingerstick devices under any circumstances.

(8) When I reuse injection equipment, there is no danger to the patient.

1. Strongly Agree
2. Agree
3. Disagree
4. Strongly disagree

(9) Do any of your colleagues reuse a syringe after changing the needle?

1. They often do this.
2. Some individuals do this.

(C) I rarely observe this.

(D) None of my colleagues do this.

(10) Do any of your colleagues reuse a needle or a syringe when accessing an IV port?

1. They often do this.
2. Some individuals do this.

(C) I rarely observe this.

(D) None of my colleagues do this.

(11) Do any of your colleagues reuse a syringe on the same patient?

1. They often do this.
2. Some individuals do this.

(C) I rarely observe this.

(D) None of my colleagues do this.

(12) Do any of your colleagues reuse a needle on the same patient?

1. They often do this.
2. Some individuals do this.

(C) I rarely observe this.

(D) None of my colleagues do this.

(13) Do any of your colleagues reuse a needle or syringe after rinsing or soaking it in warm water?

1. They often do this.
2. Some individuals do this.

(C) I rarely observe this.

(D) None of my colleagues do this.

(14) Do any of your colleagues reuse a needle or syringe after heat sterilization?

1. They often do this.
2. Some individuals do this.

(C) I rarely observe this.

(D) None of my colleagues do this.

(15) Do any of your colleagues reuse a fingerstick device?

1. Usually they reuse fingerstick devices after wiping or rinsing off visible blood.
2. They rarely reuse fingerstick devices after wiping or rinsing off visible blood.
3. They never reuse fingerstick devices without cleaning them with a disinfectant effective against HIV, HBV and HCV or a 1:10 bleach solution.
4. They never reuse fingerstick devices under any circumstances.

Questions for hospitals:

1. Did this hospital participate in the Making Medical Injections Safer project? ( Y / N )

(ask if in South Africa)

1. Are infection control guidelines posted in the hospital? ( Y / N )
2. How do you sterilize your sutures and other reusable supplies? List all processes:

________________________________________________________________________________________________________________________________________________________________________________________________________________________________________________________________________________________________

1. How does the hospital dispose of sharps waste?

________________________________________________________________________________________________________________________________________________________________________________________________________________________________________________________________________________________________

1. Supplies ordered January 1, 2009-December 31, 2009:
2. Number of syringes:
3. Number of needles:
4. Number of syringes + needles (detachable):
5. Number of syringes + needles (fixed):
6. Number of catheters:
7. Number of saline bags:
8. Number of safety boxes:

**Patient Safety Assessment Informed Consent**

Contact information for the principal investigator:

Hello. We are conducting research patient safety and adherence to the universal precautions in your hospital. We would appreciate your participation in this study, as it will assist us in improving the quality of health care services. We would like to interview you about common medical procedures you perform and sterilization routines at your hospital.

We do not anticipate the study will present any medical or social risk to you. We will ask you questions about your own personal practices. The information we gather will be reported in anonymous form, and personally identifying information will not be collected. Our findings will be reported in the public health literature and to medical professionals working in the community and to public health officials.

If you want to withdraw from the study at any time you may do so without penalty. The information on you up to that point would be destroyed.

Once the study is completed, we will publish and present the results to researchers and to the Ministry of Health. In the meantime, if you have any questions, please ask us or contact the principal investigator. If you would like to know the results of the study upon completion, please again contact the principal investigator.

I _________________ indicate by signing for the study participant that this volunteer has read and understood the information contained in this letter, and has agreed to participate in the study, on the understanding that he or she may withdraw during the data collection period and may refuse to answer any questions.

Name _______________________________Date ___________________

(Signature)

1. Khamassi S, Oniang’o R, Bisika T, Pieper C, Athembo P, Asres G, Durojaye E, Paulino K, Mfinanga S, Irunde H, Jagun S, Kip E, Saoke P, Mehari E, Makadzange P, Macauley A, Okwen M, Kalyesubula I, Morar A, Chenya E, Masembe V, Kasongo K, Byamugisha C, Nyasulu D, Reid U, Billimoria H, Gisselquist D, Reid S. Unsafe health care in Africa: a joint statement of the research agenda. *International Journal of STD & AIDS* 2009; 20: 879-880. [↑](#footnote-ref-2)
2. Perz J, Thompson N, Schaefer M, Patel P. U.S. Outbreak investigations highlight the need for safe injection practices and basic infection control. *Clinics in Liver Disease* 2010; 14: 137-151. [↑](#footnote-ref-3)
3. Reid S. Increase in clinical prevalence of AIDS implies increase in unsafe medical injections. *International Journal of STD & AIDS* 2009; 20: 295-299. [↑](#footnote-ref-4)
4. Reid S, Juma O. Minimum infective dose of HIV for parenteral dosimetry. *International Journal of STD & AIDS* 2009; 20: 828-833. [↑](#footnote-ref-5)
5. Reid S, Dawad S, Van Niekerk A. Iatrogenic HIV transmission in South Africa: Evidence, estimates, and moral perspectives. *South African Family Practice* 2010; in press. [↑](#footnote-ref-6)
6. Emmanuel F. Outbreak investigation: Mohalla JogiPura, Jalal Pur Jattan. HIV/AIDS Surveillance Project. [↑](#footnote-ref-7)
7. Safe Injection Practices Coalition. One needle, one syringe, only ONE time. *PRNewswire* May 26, 2010. [↑](#footnote-ref-8)
8. Safe Injection Global Network 10^th^ Annual Conference, Geneva, Switzerland, December 2009. [↑](#footnote-ref-9)
9. Lopman B, Garnett G, Mason P, Gregson S. Individual level injection history: a lack of association with HIV incidence in rural Zimbabwe. PLoS Med 2005; 2(2): e37 [↑](#footnote-ref-10)
10. Wawer M, Sewankambo N, Berkley S, Serwadda D, Musgrave S, Gray R, Musagara M, Stallings R, Konde-Lule J. Incidence of HIV-1 infection in a rural region of Uganda. BMJ 1994; 308(6922): 171-173 [↑](#footnote-ref-11)
11. Kiwanuka N, Gray R, Serwadda D, Li X, Sewankambo N, Kigozi G, Lutalo T, Nalugoda F, Wawer M. The incidence of HIV-1 associated with injections and transfusions in a prospective cohort, Rakai, Uganda. AIDS 2004; 18(2): 342-344. [↑](#footnote-ref-12)
12. Quigley M, Morgan D, Malamba S, Mayanja B, Okongo M, Carpenter L, Whitworth J. Case-control study of risk factors for incidence HIV infection in rural Uganda. J Acquir Immune Defic Syndr 2000; 23(5): 418-425 [↑](#footnote-ref-13)
13. Bulterys M, Chao A, Dushimimana A, et al. HIV transmission through health care in sub-Saharan Africa, authors’ replies [letter]. Lancet 2004; 364: 1665-1666 [↑](#footnote-ref-14)
14. Mermin J, Musinguzi J, Opio A, Kirungi W, Ekwaru J, Hladik W, Kaharuza F, Downing R, Bunnell R. Risk factors for recent HIV infection in Uganda. JAMA 2008; 300(5): 540-549 [↑](#footnote-ref-15)
15. N'Galy B, Ryder R, Bila K, Mwadagalirwa K, Colebunders R, Francis H, Mann J, Quinn T. Human immunodeficiency virus infection among employees in an African hospital. N Engl J Med 1988; 319(17): 1123-1127 [↑](#footnote-ref-16)
16. Bulterys M, Chao A, Habimana P, Dushimimana A, Nawrocki P, Saah A. Incident HIV-1 infection in a cohort of young women in Butare, Rwanda. AIDS 1994; 8(11): 1585-1591 [↑](#footnote-ref-17)
17. Quigley M, Morgan D, Malamba S, Mayanja B, Okongo M, Carpenter L, Whitworth J. Case-control study of risk factors for incidence HIV infection in rural Uganda. J Acquir Immune Defic Syndr 2000; 23(5): 418-425 [↑](#footnote-ref-18)
18. Mann J, Francis H, Quinn T, Bila K, Asila P, Bosenge N, Nzilambi N, Jansegers L, Piot P, Ruti K, et al. HIV seroprevalence among hospital workers in Kinshasa, Zaire. Lack of association with occupational exposure. JAMA 1986; 256(22): 3099-3102 [↑](#footnote-ref-19)
19. Kumwenda N, Kumwenda J, Kafulafula G, Makanani B, Taulo F, Nkhoma C, Li Q, Taha T. HIV-1 incidence among women of reproductive age in Malawi. Int J STD AIDS 2008; 19: 339-341 [↑](#footnote-ref-20)
20. Whitworth J, Biraro S, Shafer L, Morison L, Quigley M, White R, Mayaja B, Ruberantwari A, Van der Paal L. HIV incidence and recent injections among adults in rural southwestern Uganda. AIDS 2007; 21(8): 1056-1058 [↑](#footnote-ref-21)
21. Todd J, Grosskurth H, Changalucha J, Obasi A, Mosha F, Balira R, Orroth K, Hugonnet S, Pujades M, Ross D, Gavyole A, Mabey D, Hayes R. Risk factors influencing HIV infection incidence in a rural African population: a nested case-control study. J Infect Dis 2006; 193(3): 458-466 [↑](#footnote-ref-22)
22. Watson-Jones D, Baisley K, Weiss H, Tanton C, Changalucha J, Everett D, Chirwa T, Ross D, Clayton T, Hayes R. Risk factors for HIV incidence in women participating in an HSV suppressive treatment trial in Tanzania. AIDS 2009; 23(3): 415-422 [↑](#footnote-ref-23)
23. Peters E, Brewer D, Udonwa N, Jombo G, Essien O, Umoh V, Otu A, Eduwem D, Potterat J. Diverse blood exposures associated with incident HIV infection in Calabar, Nigeria. *Int J STD AIDS* 2009; **20**:846-851. [↑](#footnote-ref-24)
24. Brewer, D. D., Roberts, J. M., Jr., & Potterat, J. J.  Punctures during prenatal care associated with prevalent HIV infection in sub-Saharan African women. Presentation at the 17th meeting of the International Society for Sexually Transmitted Diseases Research, Seattle, July, 2007. [↑](#footnote-ref-25)
25. Shields J. Patient-to-patient transmission of HIV. *Lancet* 1994; 343: 415-416. [↑](#footnote-ref-26)
