# Supplementary material for: Uncovering high rates of unsafe injection equipment reuse in rural Cameroon: validation of a survey instrument that probes for specific misconceptions
Source: Harm Reduct J. 2011 Feb 7;8:4. doi: 10.1186/1477-7517-8-4 (PMC3041680; doi:10.1186/1477-7517-8-4)
Supplement: Additional file 2 — A policy document from the Tanzania Food and Drugs Authority. It describes the new injection safety policy in Tanzania, restricting the importation of syringes that do not have reuse prevention features that engage automatically. [file 1477-7517-8-4-S2.DOC]

**PUBLIC NOTICE**


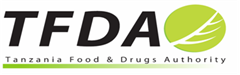


**PHASING OUT AND LIMITING USE OF STANDARD DISPOSABLE SYRINGES IN FAVOUR OF AUTODISABLE SYRINGES**

In support of the Ministry of Health and Social Welfare policy on safe injections and healthcare waste management and in order to protect safety of patients and embrace new technology, TFDA convened a stakeholders meeting on 1^st^ September, 2009 in which recommendations for phasing out and limiting use of standard disposable syringes in favour of auto disable syringes (AD) were discussed and agreed upon.

AD syringes are a type of re-use prevention syringes which disable automatically after use. Besides AD syringes there are other re-use prevention types which have to be disabled voluntarily after use.

Further to the agreement reached in September 2009, TFDA hereby announces that with effect from **1^st^ May 2010** it will no longer issue permit for importation of standard disposable and voluntarily disabling re-use prevention syringes of 10ml or less, unless these are meant for specific procedures like naso-gastric feeding, blood drawing and delicate aspirations. Guidance on importation of these exceptional quantities will be issued by TFDA.

Note that syringes of more than 10ml may still be imported without additional restrictions till further notice.

**To ensure smooth transitions and success of this policy change, it was agreed that a nine (9) months grace period be given to all affected individuals and companies to finalize the phasing out of standard disposable syringes and switch over to auto-disable syringes. This period has now been set to be from 1^st^ May 2010 to 31^st^ January 2011.**

Henceforth import authorization for AD syringes shall be granted only if the product has been pre-qualified by World Health Organization and registered by TFDA.

Prospective importers are advised to seek further guidance on acceptable quality standards of AD syringes and any other information related to this circular from TFDA.

Issued by:

**Director General**

**Tanzania Food and Drugs Authority (TFDA)**

**Mandela Road, External Mabibo**

**P.O. Box 77150**

**Dar es Salaam**

**Tel: +255 22 2450 512/2450 751/2452108**

**Fax: +255 22 2450793**

**Email:** [**info@tfda.or.tz**](mailto:info@tfda.or.tz)**, website:** [**www.tfda.or.tz**](http://www.tfda.or.tz/)
