# Supplementary material for: Uncovering high rates of unsafe injection equipment reuse in rural Cameroon: validation of a survey instrument that probes for specific misconceptions
Source: Harm Reduct J. 2011 Feb 7;8:4. doi: 10.1186/1477-7517-8-4 (PMC3041680; doi:10.1186/1477-7517-8-4)
Supplement: Additional file 3 — Hospital corruption in Cameroon. A copy of a newsletter that details the findings and recommendations of recent investigations into hospital corruption in Cameroon. [file 1477-7517-8-4-S3.PDF]

ECLAIRAGES - LIGHTINGS

Strategic Health Information **Bulletin** d'Informations Sanitaires Stratégiques - **Cameroon**

Editorial

Ce numéro d'Eclairages vous introduit aux concepts de gouvernance et de participation des parties prenantes à la viabilisation des districts de santé au Cameroun au moment où le processus de décentralisation passe à la phase opérationnelle. En effet, si la démarche de déconcentration était engagée depuis l'introduction de l'approche District accompagnée de la mise en place des structures de dialogue, il demeure vrai que la place, le rôle et les responsabilités des divers acteurs sont restés flous notamment en termes d'attributions des acteurs, de contrôle social et de transparence. Bonne Lecture!

Editorial

This issue of Lightings introduces concepts on governance and stakeholders engagement in the process of health district development in Cameroon while decentralization legislation is getting into its operational stage. If the deconcentration process has been implemented for a while in our health sector with the introduction of health districts accompanied by the establishment of dialogue structures, one should acknowledge the persistent need for clarity in defining the role and responsibilities of diverse actors namely in terms of accountability and need for transparency. Enjoy your Bulletin!

Vol II N°1 Jan 2010

Focus 1
Hospital and **Corruption** et Hôpital 2
Quid 2
Improving Health District Governance 3
Améliorer la Gouvernance dans le District 3
Actu CDBPS - CDBPH News 4

GOUVERNANCE EN SANTÉ **FOCUS** HEALTH GOVERNANCE

La gouvernance en santé est considérée comme l'ensemble des règles, procédures et comportements qui affectent la façon dont les pouvoirs sont exercés, surtout en ce qui concerne l'autorité, la responsabilité, l'ouverture, la participation, l'efficacité et la cohérence pour atteindre les objectifs du système de santé et améliorer l'état de santé des populations.

En 2007, la mauvaise gouvernance a été identifiée comme une des raisons de l'incapacité du Cameroun à atteindre les objectifs du millénaire pour le développement (OMD) liés à la santé. Plusieurs acteurs interrogés lors de l'évaluation à mi-parcours de la Stratégie Sectorielle de Santé (SSS) ont décrié la mauvaise gouvernance dans les services de santé de district et les hôpitaux. En effet, selon les rapports de supervision, les budgets adoptés pour les services de santé de district ou les hôpitaux de district ne sont pas publiés; peu nombreux sont les comités de santé et de gestion qui tiennent les quatre réunions statutaires annuelles; les mandats des membres sont rarement renouvelés à temps, et l'engagement de l'ensemble des acteurs de la santé est mitigé au sein des différents comités.

Selon le Concours Qualité 2007, les composantes de gouvernance enregistrent les plus mauvais résultats. Seuls quelques districts ont renseigné les informations financières requises notamment celles issues du recouvrement des coûts. La participation à la planification, la transparence dans la passation des marchés, la prise en compte de la voix des personnels et des communautés, le sens des responsabilités des gestionnaires sont faiblement valorisés. Moins de 35 districts font mention de bonnes pratiques de gouvernance, surtout celles relatives aux mesures incitatives pour la gouvernance et l'éthique, l'engagement communautaire (5%), le suivi et l'évaluation (13%), les mécanismes de partage des risques et les fonds d'indigence (5%). La mercantilisation du secteur de la santé se renforce malgré le taux de pauvreté de la population à 40%, les fonds de solidarité pour les indigents ne sont pas opérationnels ou manquent de procédures transparentes dans de nombreux districts. Les conflits d'intérêts non déclarés et les accords délictueux détériorent la qualité des échanges au sein des comités de santé et de gestion; une étude réalisée par GTZ dans les années 1990 a relevé que le taux de « grignotage » du budget national alloué à un district pouvait atteindre 40%. Malgré la réglementation sur les gratifications au personnel en service dans les hôpitaux selon les revenus générés localement, le personnel de la santé continue d'exprimer sa frustration devant le manque de transparence dans l'allocation et le partage des quoteparts et gratifications. En 2006, Transparency International a classé le secteur de la santé au 9e rang des 20 secteurs les plus corrompus au Cameroun. La SSS actualisée 2001- 2015 en vue d'atteindre les OMD, fixe comme priorités l'amélioration de la gouvernance dans les districts de santé et le renforcement du système de district de santé. Ce numéro d'Eclairages tente d'apporter quelques pistes pour y parvenir.

Health governance is the core of rules, processes and behaviours that affect the way in which powers are exercised, particularly with regard to authority, accountability, openness, participation, effectiveness and coherence to achieve health system outcomes and to improve people health status.

In 2007, poor governance was identified as one reason of the inability for Cameroon to achieve the health related MDGs. Many stakeholders interviewed during the midterm evaluation of the Health Sector Strategy (HSS) repeatedly reported poor governance in districts and health facilities. Indeed, according to supervisory reports, adopted budgets for district services and hospitals are not publicised; few health committees, district services and hospital management boards reported holding quarterly meetings as per requirement; membership is rarely renewed in due time and involvement of the full array of health stakeholders in health committees is mitigated.

According to the 2007 SQI Assessment, governance components scored the worst. Financial data requested, especially district incomes from user fees, were frequently missing. Participation into planning, transparency in procurement process, personnel voice, community voice, accountability of the district management team, were poor. Less than 35 districts refer to governance best practices, especially concerning the incentives for governance and ethics, community involvement (5%), monitoring and evaluation (13%), the risk-sharing mechanisms and equity funds (5%). The commercialization of the health sector is stronger despite the 40% rate of poverty; equity funds to support provision of care to indigent people are not operating in many districts or lack transparent procedures. Non-declared conflicts of interests and tortuous agreements deteriorate the quality of the discussions between the members of health committees and management boards; a survey by GTZ in the 1990's hinted that the rate of "grazing" of the central budget allocated to a district can reach 40%. Despite the provision of the regulation on bonuses to be paid to health professionals in hospitals according to locally generated incomes, the health personnel continues to express frustration with regard to lack of transparency in sharing mechanisms of these benefits and bonuses. In 2006, Transparency International classified the health sector at the 9th rank among the 20 sectors most concerned by corruption in Cameroon. The revised 2001-2015 HSS to achieve health related MDGs sets bettering health district governance and health district system strengthening as top priorities. This issue of Lightings tentatively proposes some ideas for achieving this objectives.

CORRUPTION À L'HÔPITAL PUBLIC **FOCUS** CORRUPTION IN HOSPITALS

En 2006, Transparency International a classé le secteur de la santé au 9e rang des 20 secteurs les plus touchés par la corruption au Cameroun. En effet, les procédures de passation des marchés au sein des hôpitaux sont longues et assez opaques, la surfacturation est presque la règle, et les conflits d'intérêts entre gestionnaires d'hôpitaux et les présidents des comités de gestion sont fréquents. La relation soignant-patient n'est pas épargnée par le racket, extorsion par menace, violence ou chantage; les paiements non officiels « K direct », « Narcose »- ECAM II a révélé que la forme de corruption la plus fréquente dans le secteur de la santé était le paiement forcé de frais non officiels sous forme de factures non enregistrées ou de facturations supplémentaires - pour des services réels ou fictifs - sous prétexte de rapidité ou qualité, paiements pour des services officiellement gratuits ou subventionnés comme les moustiquaires ou le dépistage du VIH, délivrance de faux certificats médicaux. Selon l'enquête RSM, 10% des consultations sont payées directement au personnel et non à la caisse, et le coût de ces factures est en moyenne de 45% supérieur aux prix indiqués.

La pratique en clientèle privée non-officielle, qui entraîne une perte de revenus à la formation sanitaire, constitue cependant une source non négligeable de revenus pour le personnel. Elle contribue à « détourner » les malades des hôpitaux publics vers les établissements privés formels ou informels. Autres formes de corruption: la recours à la fibre ethno communautaire, familiale ou amicale; le responsable détient le pouvoir discrétionnaire d'accorder des privilèges à certains patients, assistés sociaux, mais aussi amis, collègues, parenté tribale etc. La corruption devient de fait une pratique sociale et naturelle acceptée par certains.

L'hôpital public est ainsi un lieu de rencontre commerciale où offre et demande sont en inadéquation. Selon une enquête de l'INS, 44,9% des patients soignés dans les cliniques privées y sont allés en raison de l'absence de praticiens dans les hôpitaux publics. L'absentéisme, indicateur de la démotivation des personnels fonctionnaires depuis la baisse de leur pouvoir d'achat dans les années 1990, trouve racine dans la quête de compensation en dehors de l'hôpital public au profit des cliniques et cabinets de soins privés. La pénurie du soignant à l'hôpital public contraint ainsi les patients à proposer des paiements supplémentaires au personnel présent dans l'espoir de bénéficier d'une prise en charge opportune. Les enquêtes de satisfaction des usagers des services publics révèlent que près de 12% des usagers des formations sanitaires se plaignaient en 2004 du marchandage des services et soins. Le pourcentage des usagers dénonçant la corruption variait de 21,9%, 17,8%, 13,1% et 3% respectivement dans les hôpitaux nationaux et régionaux, les hôpitaux de district, les CMA et les CSI.

Les causes sont multiformes: la présence sans cesse croissante de personnels à statut précaire plus assidu que le personnel régulier malgré leur salaire dérisoire, la faible circulation de l'information entre parties prenantes avec avantage aux soignants, la « peur de mourir » qui altère le contrat entre malade et soignant d'une obligation de moyens à une obligation de résultats espérée et souhaitée par le malade et quelquefois « promise » par le soignant.

In 2006, Transparency International classified the health sector at the 9th rank among the 20 sectors most concerned by corruption in Cameroon. Indeed, contracting and procurement procedures in hospitals are lengthy and not very transparent, over-invoicing are almost the rule and conflicts of interests are frequent among top executives of hospitals and chairs of management. The caregiver-patient relationship is facing several unethical practices: racketing – extortion through threats, violence or blackmail – nonofficial payments « the small thing » or « grease » – the second Household Survey [10] has revealed that the major form of corruption in the health sector was the forced payment of non statutory fees – unregistered billing – extra-billing of real or presumed complementary services for their supposed rapidity or quality – payment for services which in fact have not been rendered – payments for services that are officially free of charge or subsidized such as mosquito nets and HIV screening and, the issuing of fake medical certificates. According to RSM survey, 10% of the consultations are paid directly to the personnel and not to the cashier and the average cost of such bills is 45% higher than the official prices.

The non-official private practice, dual practice, causes a loss of revenues for the health facility but constitutes a very source of revenues for the personnel. It contributes to "deflect" public hospital patients towards formal or informal private institutions. Other types are tribal and community-based clientelism, favouritism and cronyism from the manager of the health facility. Corruption hence become a socially and culturally acceptable practice.

State own health facility has become a commercial field where experimentation of the law of offer and demand occurs daily. According to the National Institute of Statistics, 44.9% of patients consulting in private clinics went there because of the absence of caregivers in State facilities. Absenteeism, an indicator of the level of motivation of civil servants since the loss of incomes in the 1990s, find its roots in the quest of financial compensation by many civil servants out of the hospital to consult in private clinics and health centres. Scarcity of caregivers in a state hospital forced patients to propose additional payments to health professionals present in the hope to be consulted more promptly or to received the timely and needed care. Satisfaction surveys of users by the National Institute of Statistics in 2004 revealed 12% of users voicing trafficking and merchandising around healthcare and health services. When asked for experiences of corruption in health facilities, the percentage respectively varies from 21.9%, 17.8%, 13.1% and 3% in National and regional hospitals, district hospitals, CMA and Health centres.

Causes are (i) increase proportion of temporary staff with poor pay roll usually more present than the regular civil servant, (ii) poor level of information sharing and users' poor literacy in health matters, (iii) fear of death that deters the contract between the caregiver and the patient from mandatory contract of means to an expected results-based contract or falsely promised by the caregiver.

EVIDENCE-INFORMED DECISION MAKING **QUID** PRISE DE DECISION ECLAIREE

La prise de décision éclairée est une approche de la pratique de gestion des systèmes et des organisations de santé qui consacre le recours systématique et transparent aux données probantes ou bases factuelles comme intrant dans la prise de décision. Elle repose sur l'application des principes de la pratique clinique fondée sur les preuves dans les domaines de la santé publique, de l'organisation des soins et services de santé. Les données probantes proviennent des revues systématiques et des informations sanitaires et enquêtes populationnelles. Les données probantes ou bases factuelles doivent faire l'objet d'une évaluation critique prenant en compte notamment les effets des interventions, leur adaptabilité, leur faisabilité autant que les considérations d'équité.

Evidence informed decision making is an approach to manage health systems and healthcare organizations that valued the systematic and transparent reference to research evidence as an input for decision making. This approach basically derives from the application of basics and concepts of evidence based medicine into public health and health services.

Evidence is provided by systematic reviews and routine health information systems and surveys. The evidence should be critically appraise in a systematic and transparent manner with regard to the effectiveness of interventions, adaptability and applicability to a specific context as well as for equity considerations.

IMPROVING GOVERNANCE FOR HEALTH DISTRICT DEVELOPMENT

In spite of the existence of a legal and regulatory framework to make governance more efficient in health districts since the Policy Declaration Reorienting Primary Health Care (1995) and the implementation of the 2001-2010 Health Sector Strategy (HSS), health district performance is still slow and district governance practices observed are neither satisfactory nor in conformity with the texts. Corruption within district hospitals is constantly voiced by users and excludes many patients from timely access to care.

The revised 2001-2015 HSS to achieve health related Millennium Development Goals (MDGs) sets bettering health district governance and health district system strengthening as priorities and, a health SWAp will support its implementation. Still there are no clear options on ways to bringing about the change in terms of health district governance.

Strategic options

1. Community empowerment to improve social accountability through a greater access to health related information; capacity building to engage local health development agents - politicians, municipal counsellors, community groups, CSOs and NGOs, traditional rulers– into priority setting, planning, budgeting, monitoring and evaluating the implementation of health initiatives; and capacity strengthening activities in leadership and health promotion amongst stakeholders.

2. Reinforcing fight against corruption in hospitals through the provision of resources for the establishment and operation of anticorruption committees within health facilities; building capacities for members of these committees to fulfil their mission; and improving community-based health insurance coverage thus reducing the level of direct payments during illnesses episodes and opportunities of coping corruption in hospitals.

3. Updating the legal and regulatory framework for health district governance to adapt to the evolving environment and new challenges related to the ongoing decentralization reform.

Implementation considerations

Due to specific evidence on effectiveness of change strategies in LMIC, it would be pertinent to adapt options 1 and 2 to local context building on the existing infrastructure and with local leadership. All the options need sound communication strategies to raise awareness amongst stakeholders both health professionals and community representatives as well as education and training activities to build capacity in field such as leadership, organizing meetings, transparent and democratic procedures. As appropriate monitoring and evaluation framework is needed for a continuing learning by doing process in every district and hospital.

Community empowerment strategies should focus on existing constituencies (municipal councils, village development associations, women associations and other community-based associations) so as to enhance to contribution to health promotion in general and foster their participation in health committees and boards. Developing leadership and management skills among district managers as well as establishing ethics committee for health professionals will also enable bringing about change.

AMELIORER LA GOUVERNANCE POUR LA VIABILISATION DU DISTRICT DE SANTE

Malgré le cadre juridique et réglementaire destiné à rendre la gouvernance plus efficace dans les districts de santé, la Déclaration de politique portant réorientation des soins de santé primaires1-995 et la mise en œuvre de la stratégie sectorielle de santé (SSS) 2001- 10, les performances des districts de santé restent encore faibles et la gouvernance des districts demeure non satisfaisante et non conforme aux textes. La corruption au sein des hôpitaux est constamment décriée par les usagers car elle empêche de nombreux malades d'accéder aux soins de santé en temps opportun.

La SSS actualisée 2001- 2015 en vue d'atteindre les Objectifs du Millénaire pour le Développement fixe comme priorités l'amélioration de la gouvernance et le renforcement du système de santé de district à travers un SWAp santé destiné à soutenir sa mise en œuvre. Voici quelques options stratégiques susceptibles de guider cette réforme nécessaire.

Options stratégiques

1. Renforcer les capacités des communautés pour améliorer le contrôle social et le sens des responsabilités des gestionnaires à travers à un meilleur accès aux informations relatives à la santé; la formation des acteurs du développement local (élus locaux, élites, associations communautaires, OSC, ONG, chefs traditionnels) à s'engager dans la promotion de la santé et à participer à la planification, budgétisation, suivi et évaluation de la mise en œuvre des interventions sanitaires dans leur territoire de compétence; et le renforcement des capacités des acteurs en leadership et en promotion de la santé.

2. Renforcer la lutte contre la corruption dans les services de santé et hôpitaux de district en allouant les ressources nécessaires à la création et au fonctionnement des comités de lutte contre la corruption; en renforçant les capacités des membres desdits comités pour l'accomplissement de leur mission. Les activités de prévention ciblée et de lutte contre la corruption de survie ainsi que la gestion des conflits d'intérêts sont nécessaires.

3. Actualiser le cadre organique, juridique et réglementaire de la gouvernance des districts de santé afin de s'adapter à l'environnement changeant (SWAp, Stratégie Sectorielle de Santé actualisée etc.) et aux nouveaux enjeux liés à la réforme de la décentralisation en cours.

Considérations de mise en œuvre

À la lecture des bases factuelles relatives à l'efficacité des stratégies de changement dans les pays en développement, la mise en œuvre des options 1 et 2 exigent une adaptation au contexte de chaque district en se fondant notamment sur l'infrastructure sociale et la structure du leadership local. Les 3 options exigent une stratégie de communication globale destinée à sensibiliser toutes les parties prenantes sur les enjeux mais aussi une stratégie d'éducation et de formation des personnels de santé et des acteurs communautaires dans les sujets tels que le leadership, l'organisation et la tenue efficace des réunions, la transparence et les approches consultatives démocratiques. Un cadre de suivi et évaluation des changements est nécessaire afin de renforcer la démarche d'apprentissage continu à partir des expériences tirées de la pratique dans chaque district et hôpital.

La capacitation des communautés exigent des actions ciblées en direction des entités déjà existantes telles que les conseils municipaux, les associations et comités de développement villageois ou d'arrondissement, les associations des femmes, les syndicats des personnels soignants, les ONG et OSC en matière de leadership et de promotion de la santé. Renforcer les capacités de leadership et de gestion des responsables des services de santé s'avèrent également nécessaire de même que la mise en place de comité d'éthique et de déontologie professionnelle.

CENTRE POUR LE DEVELOPPEMENT DES BONNES PRATIQUES EN SANTE
HOPITAL CENTRAL DE YAOUNDE

Avenue Henry Dunant - Messa Yaoundé 2
BP 87 Yaoundé - Cameroun

Phone: + 237 220 819 19
Fax: +237 222 220 86
Email : cdbpsh@yahoo.fr

www.cdbph.org

EN SAVOIR PLUS **FURTHER READING

From the Ministry of Public Health
(2009) Revised Health Sector Strategy 2001-2015.
(2008) Final Report of the Systemic Quality Improvement Assessment
(2007) Midterm Evaluation Report 2001-10 Health Sector Strategy.
(2006) Survey on Determinants of Service Utilization and Access to Medicines
(1996) The Framework Law N° 96/03 Orienting the Health Sector
Vinard J (2007). Report on Strategy to Fight against Corruption and Governance Indicators within the Health Sector in Cameroon.
Ambegaokar M, Ongolo-Zogo P, Aly T et al. (2004). Team Work and Incentives in the Cameroon Health Sector. Research Report.
Balique H. (2003). Situational Analysis for Hospital Reform in Cameroon. Consultation Report.
From the National Institute for Statistics
2009 Household Survey III - 2001 Household Survey II
2006 Public Services' Users Satisfaction Survey
Transparency International. Annual Report – Cameroon 2006
Scientific papers
Bossert T. (1998). Analysing the decentralization of health systems in developing countries: decision space, innovation and performance. Social Sciences and Medicine, 47:1513-1527
Báez C, Barron P. (2006). Community voice and role in district health systems in east and southern Africa: A literature review. Equinet Discussion Paper 39. June 2006.
Levers LL, Magweva FI, Mpofu E. A literature review of district health systems in east and southern Africa: Facilitators and barriers to participation in health. Equinet Discussion Paper 40
Loewenson R (1998). Community participation in health systems and their contribution towards health and health systems governance; processes and structure. Institute of Development Studies IDS.
Vega-Romero R, Tovar MT (2007). The role of civil society in building an equitable health system. Paper prepared for the Health Systems Knowledge Network of the WHO Commission on the Social Determinants of Health. June 2007.
Wallerstein N (2006). What is the evidence on effectiveness of empowerment to improve health? Copenhagen, WHO Regional Office for Europe (Health Evidence Network report; http://www.euro.who.int/Document/E88086.pdf accessed 19 April 2010
NICE. Community engagement: review 1 - social determinants of health (2009). http://www.nice.org.uk/nicemedia/live/11929/44494/44494.pdf accessed 19 April 2010
Vian Taryn (2008). Review of corruption in the health sector: theory, methods and interventions. Health Policy and Planning 23:83-94.
Meagher P (2005). Anti-corruption agencies: rhetoric versus reality. The Journal of Policy Reform 8:69–103.

ACTU CDBPS ** CDBPH NEWS

Les 7 et 8 Décembre 2009 s'est tenu le tout premier atelier sur la prise de décision sur les bases factuelles à l'intention des responsables du Minsanté et des universitaires. La trentaine de participants a pu ainsi améliorer ses connaissances sur les ressources de bases factuelles, les démarches d'acquisition, d'évaluation, d'adaptation et d'utilisation des bases factuelles. L'atelier a également permis d'identifier quelques besoins prioritaires en recours aux bases factuelles. Cet atelier a été suivi du 9 au 11 Décembre par une session de formation à la méthodologie des revues systématiques selon l'approche COCHRANE ouverte à une vingtaine d'enseignants chercheurs qui ont ainsi acquis des connaissances sur les méta analyses, le développement d'un protocole de revue systématique, les critères de qualité et la lecture critique des articles. Les deux ateliers étaient organisés en partenariat avec le Centre Collaboratif Cochrane du Cap (Afrique du Sud).

µµµµµµµµµ

Les 25 et 26 Mars 2010, un second groupe de responsables du Minsanté et du CCAM ont été initiés à la prise de décision éclairée par les bases factuelles et le développement des bonnes pratiques en santé publique. Au cours de cet atelier, les projets de réforme pouvant bénéficier des données probantes ont été identifiés et un atelier de formation des cadres à la rédaction des notes d'information sanitaire sera organisé en juin 2010 afin de créer une masse critique de cadres au sein des directions du Minsanté.

µµµµµµµµµ

On December 7 and 8, the very first workshop on Evidence informed decision making in public health was organised for thirty participants from the Ministry of public health as well as lecturers and researchers from academia. Participants were trained on tools and resources for Evidence to Policy, methods for asking the right question, acquiring relevant evidence, critically appraising the evidence and using evidence for public health decision making. Subsequently, priority needs in evidence informed decision making and for research synthesis were identified. The workshop were organised back to back with a three day course on systematic reviews using the Cochrane Methods. Twenty participants from universities were exposed to concepts and basics of systematic reviews as well as critical appraisal of individual studies and meta analysis techniques. The facilitation of both activities were provided with the support of the South African Cochrane Collaborative Centre in Cape town.

µµµµµµµµµ

A second workshop was organised on March 25-26 for senior staff from the MoH and Cameroon Coalition Against Malaria to initiate them to Evidence to Policy and identify priority needs for research synthesis to inform some reforms already in the pipe within the health sector. It has been agreed to train two cadres in 7 directorates from the MoH so as to create a critical mass of staff able to prepare policy briefs.

Équipe de Rédaction *** Editorial Team
Dr Pierre Ongolo Zogo, Dr David Yondo, Dr François Colin Nkoa, Dr Jean Serge Ndongo, Dr Robert Marie Mba, Mme Renée Cécile Bonono-Momnougui, Dr Marie José Essi, Mr Henri Atangana Ondoa
