# Supplementary material for: Uncovering high rates of unsafe injection equipment reuse in rural Cameroon: validation of a survey instrument that probes for specific misconceptions
Source: Harm Reduct J. 2011 Feb 7;8:4. doi: 10.1186/1477-7517-8-4 (PMC3041680; doi:10.1186/1477-7517-8-4)
Supplement: Additional file 4 — Questions for All Injection Providers. An expanded injection safety assessment questionnaire informed by infection control compliance research that explores the reasons for unsafe injection equipment reuse. [file 1477-7517-8-4-S4.DOC]

Questions for all injection providers

(1) Did you receive pre-service training in injection safety?

1. Yes, and I feel I have all the preparation I need
2. Yes, but I feel I need more training in this area
3. No, but I do not feel pre-service training is necessary
4. No, and I feel I needed pre-service training in this area

(2) Did you receive in-service training in injection safety?

1. Yes, and I feel I have all the preparation I need
2. Yes, but I feel I need more training in this area
3. No, but I do not feel in-service training is necessary
4. No, and I feel I need training in this area

(3) Have most of your coworkers been trained in injection safety?

1. Yes, and I feel they have all the preparation they need
2. Yes, but I feel they need more training in this area
3. No, but I do not feel specialized training is necessary
4. No, and I feel they need training in this area

(4) Does your facility’s infection control team give you clear guidance on injection safety?

1. Yes, and I feel compliance with that guidance is high.
2. Yes, but I feel compliance with that guidance could be higher.
3. No, but I do not feel I need special guidance on injection safety.
4. No, and I feel clear guidance on injection safety is needed at my facility.

(5) Is your ward supplied with matching quantities of needles and syringes?

1. Yes, at all times
2. Yes, most times
3. No, the ward is supplied with more needles than syringes most times
4. No, the ward is supplied with more needles than syringes at all times

(6) Is injection equipment loose in the ward or is it removed from a new, sealed package for the patient to see for every injection?

1. Loose in the ward at most times
2. Loose in the ward some times
3. Always in a sealed package for the injection provider to see
4. Always in a sealed package for the patient to see

(7) Are used needles and syringes always deposited directly in a sharps box?

1. Used needles and syringes always go in a sharps box immediately after an injection.
2. Used needles and syringes are always thrown away immediately, but not necessarily in a sharps box.
3. Used needles or syringes can be found loose in the ward but should not be reused.
4. Used needles or syringes are placed in rinsing pan prior to reuse.
5. Used needles or syringes are heat sterilized prior to reuse.

(8) Is there conflict over injection policies on your ward?

1. Yes, we have conflicting guidelines or directives on injection safety.
2. Yes, guidelines are not clear and practice varies from person to person.
3. Yes, we have policies with which some individuals disagree.
4. No, there is agreement on injection policies.

(9) Do you have good communication with the infection control nurses in your hospital?

1. Yes, I feel they listen to me and express their concerns respectfully.
2. Yes, but I feel their expectations don’t take my perspective into account.
3. No, I feel ignored by the infection control nurses and I don’t always know what their expectations are.
4. No, I don’t interact with the infection control nurses at all.

(10) Using a new needle and syringe for every injection is wasteful under certain circumstances.

1. Strongly agree
2. Agree
3. Disagree
4. Strongly disagree

(11) I feel bad about multiple use of needles or syringes.

1. Strongly agree
2. Agree
3. Disagree
4. Strongly disagree
5. Doesn’t apply to me

(12) I am not completely convinced of the usefulness and importance of using a new needle and syringe for every injection.

1. Strongly Agree
2. Agree
3. Disagree
4. Strongly disagree

(13) Most of my colleagues think that injection safety prescriptions should be adhered to as well as possible.

1. Strongly Agree
2. Agree
3. Disagree
4. Strongly disagree

(14) Good injection practices are appreciated in my professional environment.

1. Strongly Agree
2. Agree
3. Disagree
4. Strongly disagree

(15) Would you reuse a syringe after changing the needle?

1. I often do this.
2. I sometimes do this.
3. I rarely do this.
4. I can think of some circumstances under which I would do this.
5. I would never do this.

(16) Would you reuse a needle or a syringe when accessing an IV port?

1. I often do this.
2. I sometimes do this.
3. I rarely do this.
4. I can think of some circumstances under which I would do this.
5. I would never do this.

(17) Would you reuse a syringe on the same patient?

1. I often do this.
2. I sometimes do this.
3. I rarely do this.
4. I can think of some circumstances under which I would do this.
5. I would never do this.

(18) Would you reuse a needle on the same patient?

1. I often do this.
2. I sometimes do this.
3. I rarely do this.
4. I can think of some circumstances under which I would do this.
5. I would never do this.

(19) Would you reuse a needle or syringe after rinsing or soaking it in warm water?

1. I often do this.
2. I sometimes do this.
3. I rarely do this.
4. I can think of some circumstances under which I would do this.
5. I would never do this.

(20) Would you reuse a needle or syringe after heat sterilization?

1. I often do this.
2. I sometimes do this.
3. I rarely do this.
4. I can think of some circumstances under which I would do this.
5. I would never do this.

(21) Would you reuse a fingerstick device?

1. Usually I reuse fingerstick devices after wiping or rinsing off visible blood.
2. I rarely reuse fingerstick devices after wiping or rinsing off visible blood.
3. I never reuse fingerstick devices without cleaning them with a disinfectant effective against HIV, HBV and HCV or a 1:10 bleach solution.
4. I never reuse fingerstick devices under any circumstances.

(22) When I reuse injection equipment, there is no danger to the patient.

1. Strongly Agree
2. Agree
3. Disagree
4. Strongly disagree

(23) Do any of your colleagues reuse a syringe after changing the needle?

1. They often do this.
2. Some individuals do this.

(C) I rarely observe this.

(D) None of my colleagues do this.

(24) Do any of your colleagues reuse a needle or a syringe when accessing an IV port?

1. They often do this.
2. Some individuals do this.

(C) I rarely observe this.

(D) None of my colleagues do this.

(25) Do any of your colleagues reuse a syringe on the same patient?

1. They often do this.
2. Some individuals do this.

(C) I rarely observe this.

(D) None of my colleagues do this.

(26) Do any of your colleagues reuse a needle on the same patient?

1. They often do this.
2. Some individuals do this.

(C) I rarely observe this.

(D) None of my colleagues do this.

(27) Do any of your colleagues reuse a needle or syringe after rinsing or soaking it in warm water?

1. They often do this.
2. Some individuals do this.

(C) I rarely observe this.

(D) None of my colleagues do this.

(28) Do any of your colleagues reuse a needle or syringe after heat sterilization?

1. They often do this.
2. Some individuals do this.

(C) I rarely observe this.

(D) None of my colleagues do this.

(29) Do any of your colleagues reuse a fingerstick device?

1. Usually they reuse fingerstick devices after wiping or rinsing off visible blood.
2. They rarely reuse fingerstick devices after wiping or rinsing off visible blood.
3. They never reuse fingerstick devices without cleaning them with a disinfectant effective against HIV, HBV and HCV or a 1:10 bleach solution.
4. They never reuse fingerstick devices under any circumstances.

(30) I receive remarks from colleagues or superiors if I reuse injection equipment.

1. Strongly Agree
2. Agree
3. Disagree
4. Strongly disagree

(31) Most of my colleagues use a new needle and syringe for every injection >50% of the time.

1. Strongly Agree
2. Agree
3. Disagree
4. Strongly disagree

(32) Are the views of your peers on injection equipment reuse important to you?

1. Very important
2. Somewhat important
3. Not very important
4. Unimportant

(33) Are the views of your infection control supervisors on injection equipment reuse important to you?

1. Very important
2. Somewhat important
3. Not very important
4. Unimportant

(34) I would use a new needle and syringe for every injection if adequately supplied with new injection equipment.

1. Strongly Agree
2. Agree
3. Disagree
4. Strongly disagree

(35) When I administer an injection, there is backflow through the needle into the syringe even if I do not pull back the plunger of the syringe.

1. Strongly Agree
2. Agree
3. Disagree
4. Strongly disagree

(36) There is space inside a syringe where fluid can accumulate without being visible.

1. Strongly Agree
2. Agree
3. Disagree
4. Strongly disagree

(37) There is backflow through the IV tubing to the needle and syringe when an IV is accessed to deliver medication or flush the IV with saline.

1. Strongly Agree
2. Agree
3. Disagree
4. Strongly disagree

(38) There is no backflow through the IV tubing to the needle and syringe when an IV is accessed to deliver medication or flush the IV with saline if there are heparin locks or valves.

1. Strongly Agree
2. Agree
3. Disagree
4. Strongly disagree

(39) A multidose vial can be contaminated with blood when accessed with a used syringe, and this contamination can be transferred to other patients receiving medication from the same vial.

1. Strongly Agree
2. Agree
3. Disagree
4. Strongly disagree

(40) HIV can survive outside the body in blood contamination that is exposed to the air for hours.

1. Strongly Agree
2. Agree
3. Disagree
4. Strongly disagree

(41) HIV can survive inside a used syringe for days to weeks.

1. Strongly Agree
2. Agree
3. Disagree
4. Strongly disagree

(42) Numerous hepatitis B virus outbreaks have been traced to injection equipment reuse in the health sector in countries with blood-borne virus surveillance.

1. Strongly Agree
2. Agree
3. Disagree
4. Strongly disagree

(43) Numerous hepatitis C virus outbreaks have been traced to injection equipment reuse in the health sector in countries with blood-borne virus surveillance.

1. Strongly Agree
2. Agree
3. Disagree
4. Strongly disagree

(44) Several HIV outbreaks in children with HIV negative mothers have been linked to injection equipment reuse in the health sector in countries with inadequate infection control practices.

1. Strongly Agree
2. Agree
3. Disagree
4. Strongly disagree

(45) Secondary infections in patients are preventable in most cases.

1. Strongly Agree
2. Agree
3. Disagree
4. Strongly disagree

(46) I have the ability to take the necessary steps to reduce the risk of secondary infections.

1. Strongly Agree
2. Agree
3. Disagree
4. Strongly disagree

(47) If one of my colleagues makes a medical error, this will be concealed from our supervisors.

1. Strongly Agree
2. Agree
3. Disagree
4. Strongly disagree

(48) If one of my supervisors becomes aware of a medical error, the unsafe practice is discussed by the whole team and steps are taken to prevent future errors.

1. Strongly Agree
2. Agree
3. Disagree
4. Strongly disagree

(49) If one of my supervisors becomes aware of a medical error, the response will be to blame the person who made a mistake and censure that individual.

1. Strongly Agree
2. Agree
3. Disagree
4. Strongly disagree

(50) When one of my patients develops a preventable secondary infection, my colleagues and I reflect on how this may have happened in order to identify any mistakes that may have been made.

1. Strongly Agree
2. Agree
3. Disagree
4. Strongly disagree

(51) The quality of care my patients receive is a priority in my workplace.

1. Strongly Agree
2. Agree
3. Disagree
4. Strongly disagree

(52) Patients’ expectations about the services they receive at this facility are unrealistic or unreasonable due to resource constraints.

1. Strongly Agree
2. Agree
3. Disagree
4. Strongly disagree

(52) Patients expect an injection even if it is not absolutely necessary.

1. Strongly Agree
2. Agree
3. Disagree
4. Strongly disagree

(53) Patient safety is a priority in my workplace.

1. Strongly Agree
2. Agree
3. Disagree
4. Strongly disagree

(54) Patient safety concerns place an undue strain on this facility’s resources.

1. Strongly Agree
2. Agree
3. Disagree
4. Strongly disagree

(55) Cost control is a priority in my workplace.

1. Strongly Agree
2. Agree
3. Disagree
4. Strongly disagree

(56) Patient safety takes priority over cost control at this facility.

1. Strongly Agree
2. Agree
3. Disagree
4. Strongly disagree

(57) Patient safety takes priority over cost control when it is my decision.

1. Strongly Agree
2. Agree
3. Disagree
4. Strongly disagree

(58) I avoid putting my patients at risk.

1. Strongly Agree
2. Agree
3. Disagree
4. Strongly disagree

(59) Many health care services carry risk for the patient, and this is unavoidable.

1. Strongly Agree
2. Agree
3. Disagree
4. Strongly disagree

(60) There is little I can do to protect my patients from HIV.

1. Strongly Agree
2. Agree
3. Disagree
4. Strongly disagree

(61) There is little I can do to protect myself from HIV.

1. Strongly Agree
2. Agree
3. Disagree
4. Strongly disagree

(62) Today HIV is a less than deadly disease because treatment is readily available.

1. Strongly Agree
2. Agree
3. Disagree
4. Strongly disagree

(63) Sharps waste is excessive because of single use policies at this facility.

1. Strongly Agree
2. Agree
3. Disagree
4. Strongly disagree

(64) Sharps waste is handled safely at this facility.

1. Strongly Agree
2. Agree
3. Disagree
4. Strongly disagree

(65) In the past six months, how many times have you experienced a needle stick injury? ___

(66) The last time you experienced a needle stick injury, did you report it?

1. Yes
2. No

(67) The last time you experienced a needle stick injury, was the source blood tested?

1. Yes
2. No

(68) In the past six months, have you used post-exposure prophylaxis because of a needle stick injury?

1. Yes
2. No

(69) Do your colleagues report needle stick injuries consistently?

1. Every time
2. Most times
3. Sometimes
4. Not often

(70) When someone reports a needle stick injury at this facility, is the source blood tested?

1. Every time
2. Most times, otherwise post exposure prophylaxis is used
3. Not consistently, and sometimes there is no testing or post exposure prophylaxis
